# High mutation rates explain low population genetic divergence at copy-number-variable loci in *Homo sapiens*

**DOI:** 10.1038/srep43178

**Published:** 2017-02-22

**Authors:** Xin-Sheng Hu, Francis C. Yeh, Yang Hu, Li-Ting Deng, Richard A. Ennos, Xiaoyang Chen

**Affiliations:** 1 Guangdong Key Laboratory for Innovative Development and Utilization of Forest Plant Germplasm, South China Agricultural University, Guangdong 510642, China; 2 College of Forestry and Landscape Architecture, South China Agricultural University, Guangdong 510642, China; 3 Department of Renewable Resources, 751 General Service Building, University of Alberta, Edmonton, AB T6G 2H1, Canada; 4 Department of Computing Science, University of Alberta, Edmonton, AB T6G 2S4, Canada; 5 Institute of Evolutionary Biology, Ashworth Laboratories, School of Biological Sciences, University of Edinburgh, Edinburgh EH 9 3JT, United Kingdom

## Abstract

Copy-number-variable (CNV) loci differ from single nucleotide polymorphic (SNP) sites in size, mutation rate, and mechanisms of maintenance in natural populations. It is therefore hypothesized that population genetic divergence at CNV loci will differ from that found at SNP sites. Here, we test this hypothesis by analysing 856 CNV loci from the genomes of 1184 healthy individuals from 11 HapMap populations with a wide range of ancestry. The results show that population genetic divergence at the CNV loci is generally more than three times lower than at genome-wide SNP sites. Populations generally exhibit very small genetic divergence (*G*_*st*_ = 0.05 ± 0.049). The smallest divergence is among African populations (*G*_*st*_ = 0.0081 ± 0.0025), with increased divergence among non-African populations (*G*_*st*_ = 0.0217 ± 0.0109) and then among African and non-African populations (*G*_*st*_ = 0.0324 ± 0.0064). Genetic diversity is high in African populations (~0.13), low in Asian populations (~0.11), and intermediate in the remaining 11 populations. Few significant linkage disequilibria (LDs) occur between the genome-wide CNV loci. Patterns of gametic and zygotic LDs indicate the absence of epistasis among CNV loci. Mutation rate is about twice as large as the migration rate in the non-African populations, suggesting that the high mutation rates play dominant roles in producing the low population genetic divergence at CNV loci.

Understanding human population genetic structure remains important for gaining insights into human history and demography, as well as for investigating genetic diseases in relation to geography and ancestry^1^,^2^,^3^. Historically, human population divergence is assessed using approaches from a variety of disciplines including archaeology, palaeontology, linguistics, climatology and genetics. Early studies of genetic divergence were conducted by investigating the genetic variability of mitochondrial DNA and Y chromosomes^4^,^5^. Currently, genome-wide SNP sites are used to measure population differentiation (e.g., the HapMap genotype data)^6^,^7^,^8^, and to search for outlier regions that are potentially associated with geographically restricted genetic diseases^9^. Different classes of genetic markers vary widely in many important characteristics, such as their mode of inheritance (paternal, maternal, or biparental), mutation rate, and degree of selective neutrality. As a result population genetic divergence can vary depending on the class of genetic marker investigated. Copy-number-variable (CNV) loci are an important cause of genetic variation in human genomes, and give rise to differences of 4.8–9.5% in the overall length of human genomes^10^,^11^. However population genetic divergence at the genome-wide CNV loci has not been investigated in detail^12^,^13^, nor has genome-wide divergence at the CNV loci been compared with that at SNP sites.

Genetic variation at CNV loci in *Homo sapiens* and other species has been extensively reviewed from a number perspectives^12^,^14^. Topics covered include the mechanisms for generating copy number variation, natural selection on duplication and deletion variants, the impacts of demographical changes on CNV loci, associations with SNP loci, and the role of CNV loci in causing diseases^10^,^11^,^12^,^15^,^16^,^17^. At the population level, the evolutionary dynamics of CNV loci can be studied within the framework of population genetics^12^,^14^. Emerson *et al*.^18^ used an infinite-site model to investigate purifying selection on copy number variation in specific gene regions in *Drosophila melanogaster*. Sjödin and Jakobsson^12^ suggested the use of a K-allele model^19^ or a stepwise mutational model^20^ to describe the mutation process at CNV loci. The neutrality of CNV loci has also been analyzed^21^,^22^. We recently developed a three-allele model to test neutrality at CNV loci, and demonstrated selective neutrality at 856 CNV loci scored in 1184 healthy individuals from the HapMap genotype data set^23^. The evolution of these CNV loci can be essentially explained by a mutation-drift process^23^. Here, we proceed with the same dataset to investigate population genetic divergence at the genome-wide CNV loci.

In comparison with variation at SNP sites, variants at CNV loci have several distinct features. First, CNV variants often differ in length by 1kbp or more^24^,^25^, whereas SNP variants differ by a single base pair. Thus although CNV loci (~4.8~9.5% of human genomes) are much less abundant than SNP sites in human genomes, they represent an important type of chromosomal structural variation^11^. Second, more complex processes are involved in generating copy number variants, including non-allelic homologous recombination (NAHR)^26^, non-homologous end joining (NHEJ), and insertion of transposable elements (TEs)^27^,^28^. These differ dramatically from the mechanisms generating point mutation (transitions and transversions) at SNP sites. Third, the average mutation rate at CNV loci is expected to be much higher than the point mutation rates at SNP sites^29^, resulting in a much younger average age of alleles for CNV than for SNP loci in natural populations^30^. Given these differences in the properties of CNV and SNP markers, we anticipate that they will vary in their degree of population genetic divergence.

To test this hypothesis, we employ genotype data at CNV loci from the HapMap Phase III populations. This has two advantages. The first is that genetic divergence among these populations has been fully investigated at genome-wide SNP sites^31^, providing the opportunity for direct comparison with results for the genome-wide CNV loci. Analysis of CNV loci has so far only been conducted with partial HapMap Phase III populations^31^ or at a particular gene site^32^. Our result should differ from existing analysis because they will include more populations with a wider range of ancestry. Increasing the number of individuals will affect both the genetic divergence and the number of common CNV loci. The second advantage for using the HapMap dataset is that exact discrete copy numbers are available for each diploid genotype at each CNV locus^31^. Although techniques for detecting CNV loci have recently been improved, discrete copy-number genotypes at each CNV locus, which are also essential for accurate case-control association testing with CNV loci^33^, are rarely archived in publically accessible data. Furthermore, the sample sizes in previous studies at CNV loci are often too small, and hence are inappropriate for population genetic structure analysis^18^,^34^,^35^. The large sample sizes in HapMap Phase III populations means that the probabilities of making either false-positive or negative CNV calls are negligible^23^.

In this study we analyze genetic divergence at the genome-wide CNV loci and compare it with that at the genome-wide SNP sites in exactly the same populations. To further address the population genetic properties of CNV loci and reinforce our explanations of evolution at CNV loci, we test LDs at both gametic and zygotic levels among all pairs of CNV loci. We compare the patterns of gametic and zygotic LDs at CNV loci with those previously reported at SNP sites^36^,^37^. Recent theoretical studies indicate that zygotic LD is more informative than gametic LD for inferring the effects of different evolutionary forces (mating system, gene flow, selection, and genetic drift)^38^,^39^. In the absence of functional epistatic selective effects among loci, gametic LD (lower order) is always greater than the maximum zygotic LD in value. Other processes, including mating system, gene flow and genetic drift, do not change this pattern although they can generate LD (statistical associations between loci)^38^,^39^. The difference between the values of gametic LD and maximum zygotic LD can be used to infer whether epistasis exists between loci. Such differences tested previously at the genome-wide SNP sites with the HapMap Phase III populations^37^, have shown the existence of epistases among many SNP sites. Here, we also investigate this property at the genome-wide CNV loci by presuming that individual CNV loci are directly/indirectly or equally involved in fitness changes. Information from LD analyses among CNV loci helps us to view the difference in population genetic divergence between SNP and CNV loci from a different perspective. Overall our objective is to infer the roles of mutation and migration in producing human population genetic divergence at the genome-wide CNV loci by comparing the single and multilocus population genetic structure of SNP and CNV loci.

## Results

### Population genetic divergence

Maximum likelihood estimates (MLEs) of allele frequencies are summarized in Table S1. Although all CNV loci are polymorphic in the pooled population, they exhibit various levels of polymorphisms among populations (Table 1). More than 80% of CNV loci are polymorphic in African populations (ASW, LWK, MKK, and YRI), but less than 60% in non-African populations except MEX (62.38%). Three Asian populations (CHB, CHD, and JPT) have about 45% polymorphic CNV loci.

African populations have 1.84–1.90 alleles per CNV locus while Asian populations have about 1.50 alleles per CNV locus. The rest of the 11 populations have intermediate numbers of alleles per locus (*N*_*a*_ = 1.6–1.66). Similarly, African populations have high gene diversity over all CNV loci (*H*_*e*_ = ~0.13) and small standard deviations (~0.15); while Asian populations have low gene diversity (~0.11) but large standard deviations (~0.16) over all CNV loci. The rest of the 11 populations have intermediate gene diversity and standard deviations (Table 1).

**Figure 1:**
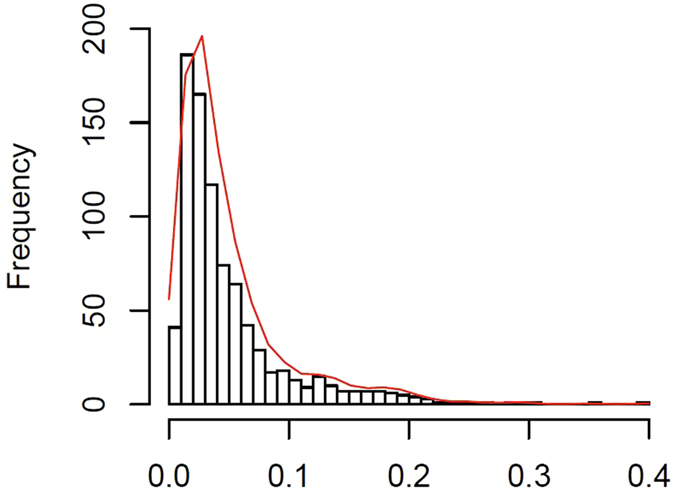
A histogram of *G*_*st*_ distribution at 856 CNV loci. The abscissa axis is the *G*_*st*_ values. The curve is based on the kernel-smoothed density function.

**Figure 2:**
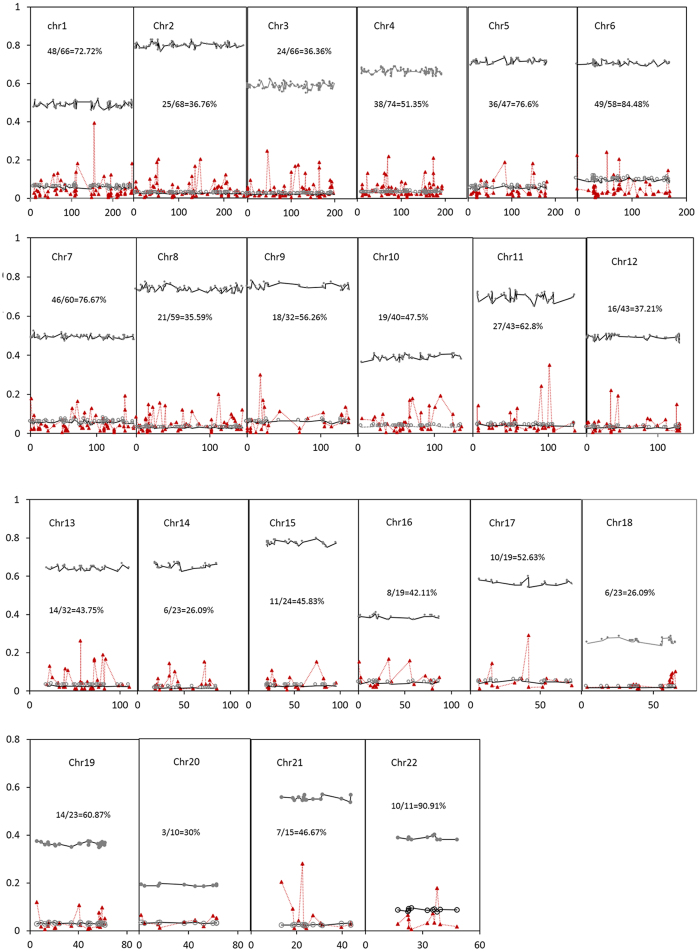
G_st_ values across chromosomes at CNV loci. The observed *G*_*st*_ values are in red, and their 95% CIs are derived from 1000 bootstrapping samples on each chromosome. The lines with opened and closed circles are the lower and upper *G*_*st*_ values of 95% CIs, respectively. The abscissa axis is the positions for CNV loci on each chromosome in Mb, and the ordinate axis is the *G*_*st*_ values.

Genetic differentiation measured by *G*_*st*_ is 0.0498 ± 0.0491 among all CNV loci, and most individual *G*_*st*_ values are around 0.05, with a few CNV loci having relatively large *G*_*st*_ values (Fig. 1). Substantial variations exist among chromosomes, especially for the small *G*_*st*_ values that are outside the 95% CIs (Fig. 2). The proportions of CNV loci exhibiting a significantly low level of population genetic divergence are 72.72% on Chr 1, 51.35% on Chr 4,76.6% on Chr 5, 84.48% on Chr 6, 76.67% on Chr 7, 56.26% on Chr 9, 62.8% on Chr 11, 52.63% on Chr 17, 60.87% on Chr 19, and 90.91% on Chr 22. The rest of the chromosomes have less than 50% of CNV loci with a significantly low level of population differentiation. None of the chromosomes has any CNV locus exhibiting a significantly high level of population differentiation (Fig. 2).

The average pairwise multilocus *G*_*st*_ ranges from 0.0038 ± 0.00001 (CHB-CHD) to 0.0421 ± 0.0001 (JPT-LWK), with the mean of 0.0255 ± 0.0114 over all pairs (Table 2). The average pairwise multilocus *G*_*st*_ in African populations ranges from 0.0059 ± 0.00001 (LWK-YRI) to 0.0128 ± 0.00002 (MKK-YRI), with the mean of 0.0081 ± 0.0025 over population pairs. The average pairwise multilocus *G*_*st*_ in non-African populations ranges from 0.0038 ± 0.00001(CHB-CHD) to 0.0352 ± 0.0001(TSI-JPT), with the mean of 0.0212 ± 0.0109 over population pairs. The average pairwise multilocus *G*_*st*_ among African and non-African populations ranges from 0.0206 ± 0.00004 (MKK-TSI) to 0.0421 ± 0.0001 (JPT-LWK), with the mean of 0.0324 ± 0.0064. over population pairs.

Compared with the pairwise multilocus *F*_*st*_ previously reported at the genome-wide SNP sites^7^, the pairwise multilocus *G*_*st*_ at the genome-wide CNV loci is generally more than three times lower (average ratio of *F*_*st(SNP)*_/*G*_*st(CNV)*_ = 3.3081 ± 1.1837; Table 2). The ratios of *F*_*st(SNP)*_/*G*_*st(CNV)*_ range from 1.3481 ± 0.0171 (LWK-YRI) to 2.1023 ± 0.0087 (MKK-LWK) in African populations, with the mean of 1.6849 ± 0.3294 over population pairs; from 0.2649 ± 0.0265 (CHB-CHD) to 3.6545 ± 0.0253 (CEU-CHD) in non-African populations, with the mean of 2.5048 ± 0.9240 over population pairs; and from 3.3.5497 ± 0.0200 (ASW-GIH) to 4.8624 ± 0.0258 (CHD-MKK) among African and non-African populations, with the mean of 4.2584 ± 0.3548 over population pairs (Table 2).

Inter-chromosomal variations in pairwise *G*_*st*_ values are substantial among different population pairs (Figure S1a), indicating the presence of differential divergences among chromosomes during the formation of populations. The pairs among African and non-African populations have large variations among chromosomes, especially on Chrs 9, 10, 16, 20, and 22 (Figure S1a), while the pairs among African populations or among non-African populations exhibit relatively stable divergences among chromosomes (e.g., CHB-JPT and CEU-CHB; Figure S1b).

**Figure 3:**
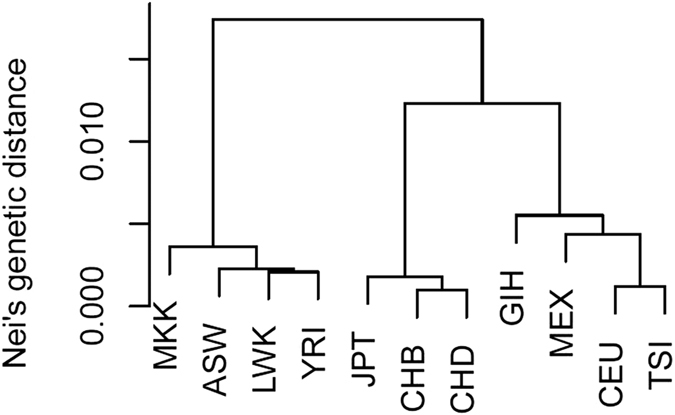
Cluster analysis of 11 human populations. The plot is based on Nei’s genetic distance by using UPGMA for hierarchical clustering.

Pairwise Nei’s genetic distances at multiple CNV loci range from 0.001 ± 0.000004 (CHB-CHD) to 0.0241 ± 0.0001 (CHD-YRI), with a mean of 0.0124 ± 0.0067 over all pairs (Table S2). The average genetic distance is 0.0029 ± 0.0010 among African populations, 0.0085 ± 0.0049 among non-African populations, and 0.0174 ± 0.0040 among African and non-African populations. Cluster analysis with the unweighted pair group method with arithmetic mean (UPGMA) shows that the three subgroups (African, Asian, and the rest of the populations) are clearly distinguished (Fig. 3). Bootstrapping resample trees (1000) using PHYLIP^40^ indicate that African and non-African populations can be separated with a probability of 100% (data not shown here).

Consider an average mutation rate of the order 10^−5^ at a CNV locus^29^, the equal effective population sizes among the 11 populations, and 25 years per generation. From the average distance 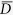 and its approximate variance *V(t*), the population isolation time is generally about *t* = 0.0124 × 5 × 10^4^ × 25 ± 0.0067 × 5 × 10^4^ × 25 = 15500 ± 8375 years among populations, *t* = 3625 ± 1250 years among African populations, about *t* = 10625 ± 6125 years among non-African populations, and about *t* = 21750 ± 5000 years among African and non-African populations.

### Gametic and zygotic LDs

Statistical tests indicate that very few pairs of CNV loci, 0.027~0.073%, exhibit significant gametic LDs in the 11 populations (Table 3; Table S3 for details). Most pairs of CNV loci have insignificant gametic LDs in each population. Among the significant gametic LDs, African populations generally have a lower proprtion of CNV locus pairs with significant gametic LDs than do most non-African populations (Table 3). The average significant r-squares are higher for CNV loci from the same chromosome (~0.76) than from different chromosomes (~0.16). Among the significant gametic LDs on the same chromosomes, more pairs come from partially overlapped CNV loci in each population (Table 3).

Patterns of gametic LDs are different among populations. African populations have more significant gametic LDs from different chromosomes than from the same chromosomes, while non-African populations except CEU and MEX have more significant gametic LDs from the same chromosomes than from different chromosomes. No common pairs of CNV loci have significant gametic LDs on different chromosomes among 11 populations, but twelve common pairs from overlapped CNV loci (except one on Chr 7) exist, with 1 on Chrs 1, 7,9,11, and 12, 3 on Chr 5, and 4 on Chr 6 (Table S4).

Tests of zygotic LDs also indicate that a very few CNV loci have significant zygotic LDs, 0 ~ 0.0359% (Table 4), which is generally less than the proportion of significant gametic LDs (Table 3). Most CNV loci with significant zygotic LDs are partially overlapped on the same chromosomes (Table S5). African populations have fewer significant zygotic LDs than do most non-African populations in significant *D*_*ij*_ (*i, j* = 0, 1, 2) except *D*_*3j*_ (j = 0, 1, 2, 3). There are twenty-two common pairs of CNV loci (mostly overlapped) of significant zygotic LDs in 11 populations, with 1 pair on Chr 1, 3 on Chr 5, 7 on Chr 6, 3 on Chr 7, 1 on Chr 9, 2 on Chr 10, 2 on Chr 11, 1 on Chr 12, 1 on Chr 13, and 1 on Chr 20 (Table 4). These locus pairs also have significant gametic LDs, while some CNV loci with significant zygotic LDs have no significant gametic LDs in 11 populations (Table S4).

For all CNV loci the maximum zygotic LD is smaller than the gametic LD in value, indicating that no epistatic effects exist between CNV loci. Both gametic and zygotic LD analyses indicate that these CNV loci are essentially in linkage equilibrium except for a few overlapped loci in each population.

### Joint migration and mutation rates

From the pairwise multilocus *G*_*st*(CNV_) (Table 2) and the pairwise multilocus *F*_*st*(SNP)_^7^,^31^, the ratios of the joint migration and nutation rates at CNV loci (*m*_*c*_ + 3*μ*_*c*_/2) to those at SNP sites (*m*_*s*_ + 2*μ*_*s*_) are estimated according to equations (9) and (12) (Table 5). The ratios range from 0.2624 ± 0.0263 (CHB-CHD) to 5.7238 ± 0.0375 (CHD-MKK), with the mean of 3.6600 ± 1.4188 over all pairs. The ratios change from 1.4126 ± 0.0144 (ASW-LWK) to 2.1402 ± 0.0088 (MKK-YRI) in African populations, with the mean of 1.6988 ± 0.3411 over population pairs; from 0.2624 ± 0.0263 (CHB-CHD) to 3.9942 ± 0.0311 (CEU-CHD) in non-African populations, with the mean of 2.6796 ± 1.0224 over population pairs; and from 3.8132 ± 0.0266 (ASW-GIH) to 5.7238 ± 0.0375 (CHD-MKK) among African and non-African populations, with the mean of 4.8157 ± 0.4929 over population pairs.

Using the average pairwise *F*_*st*(SNP)_ = 0.0956 ± 0.0567^7^,^31^ and the average pairwise *G*_*st*(CNV)_ = 0.0255 ± 0.0114 across all population pairs, we obtain (*m*_*c*_ + 3*μ*_*c*_/2)/(*m*_*s*_ + 2*μ*_*s*_) = 4.0396 ± 3.2341, where a large standard deviation arises from the variation among populations. The above estimates indicate that the joint migration and mutation rates are generally much greater at the genome-wide CNV loci than at the genome-wide SNP sites.

The ratio of the mutation rate to the migration rate at CNV loci can be approximately quantified. According to equations (13) and (14), estimates of *μ*_*c*_/*m* are summarised in Table 5, which range from 0.0352 ± 0.0177 (TSI-CEU) to 3.1492 ± 0.0250 (CHB-MEX), with a mean of 1.8153 ± 0.9016 over population pairs (except for a negative value for the CHB-CHD pair). The mutation rate is generally smaller than the migration rate among African populations (0.2392 ± 0.0115~0.7601 ± 0.0055; Table 5), but is greater than the migration rate among non-African populations (1.2036 ± 0.5881) or among African and non-African populations (2.4655 ± 0.4384).

Estimate of *μ*_*c*_/*m* is 2.0264 ± 2.1561 from the rate (*m*_*c*_ + 3*μ*_*c*_/2)/(*m*_*s*_ + 2*μ*_*s*_) = 4.0396 ± 3.2341 in the 11 populations, and 2.0352 ± 2.0909 from (*m*_*c*_ + 3*μ*_*c*_/2)/(*m*_*s*_ + 2*μ*_*s*_) = 4.0529 ± 3.1364 in four populations (CEU, YRI, CHB, and JPT)^7^,^8^ (average pairwise *F*_*st*(SNP)_ = 0.1265 ± 0.0675; average pairwise *G*_*st*(CNV)_ = 0.0354 ± 0.0158 in the present study). These estimates indicate that the mutation rate at CNV loci is generally about twice as large as the migration rate.

## Discussion

Our results indicate a closer population genetic relationship at CNV loci than at SNP sites among 11 HapMap Phase III populations. Previous reports indicate a similar pattern at specific loci among African, European and East Asian populations (HapMap Phase II data)^41^, or among HapMap Phase II populations (*F*_*st*_ = ~0.11 at the genome-wide SNP sites)^42^. A general similarity in relative population genetic structure at CNV loci and SNP sites is also reported with more populations (29) and fewer CNV loci (396) and individuals (405 in total), but the difference is not quantified^13^. LD analyses indicate that these CNV loci are essentially in linkage equilibrium except for a few overlapped loci. Epistasis does not exist for any pair of CNV loci, presuming that these CNV loci are not selectively neutral or equally additive in influencing fitness. This result is different from those at the genome-wide SNP sites where epistasis occurs among many intron SNPs^37^. The results provide additional support for a recent report indicating that the 856 CNV loci are selectively neutral in each population^23^. The evolutionary processes for the low level of population divergences are different from those at the nonsynonumous SNP sites with *F*_*st*_ < 5% where negative selection is thought to be involved^31^.

Note that our analyses are based on the three-allele system for describing the evolution at a CNV locus because the maximum number of allele copies is four in a diploid genotype. These 856 CNV loci are shown to exhibit neutrality among 1184 healthy individuals^23^. A system of more than three alleles is needed when more than four allele copies occur in a genotype at any CNV locus. This could likely occur when fewer individuals are surveyed or when unhealthy individuals are included because the number of common CNV loci could become fewer with smaller sample sizes. Under this situation, a neutrality test at CNV loci is needed for small sample sizes, and the extent of population genetic divergence could be different from the results reported here. This needs further verification.

Nei’s genetic distance at the genome-wide CNV loci is generally comparable to those between human populations at the common protein or blood group loci^43^. However, African populations have even smaller genetic divergence at CNV loci. In the process of mutation-drift at the 856 CNV loci^23^, population differentiation is expected to occur more recently owing to the high mutation rates at CNV loci. The time estimates since divergence are much shorter than those for general population genetic divergence in humans estimated from common protein loci (~120 Kyrs between human populations^43^), or than the postulated time (>100 Kyrs) for modern humans to leave Africa and colonize the rest of the world. Because the assumption for 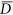 = 2 *μt*^43^,^44^ is violated due to the unequal effective population sizes among populations^45^,^46^, the varying mutation rates among loci, and the finite number of alleles at a CNV locus (not the infinite-allele model)^23^, the preceding estimates might provide a reference for the minimum divergence times.

Patterns of genetic divergence at CNV loci may reflect the historical divergence in forming modern human origins. The common pattern at both CNV and SNP loci is that the smallest genetic divergence is present among African populations, followed by among non-African populations, and then among African and non-African populations. Polymorphisms at CNV loci decrease from African to non-African populations. More alleles per CNV locus in African populations suggest a longer-term accumulation of mutants. These patterns are consistent with the Out of African model rather than with the multiregional model for modern human origins^47^,^48^. Genetic drift effects reduce genetic diversity in non-African populations. Further inferences on the evolutionary processes occurring among non-African populations would require additional information besides the comparison of polymorphisms at CNV loci. Nevertheless, the genetic relationships among non-African populations show a clear separation of Asian populations from non-Asian populations. Evidence at genome-wide CNV loci supports the hypothesis that CHB and CHD have a very close genetic relationship. This is slightly different from the genetic relationships revealed by the patterns of zygotic and gametic LDs at the genome-wide SNP sites where JPT and CHD have a very close genetic relationship^37^. Genetic drift effects could explain the relative small differentiation in polymorphism at CNV loci in Asian and European populations. Both CHB and CHD have relatively smaller genetic drift effects than JPT^45^, and hence have higher polymorphisms (1.50 vs1.48 alleles per CNV locus). CEU probably has relatively smaller genetic drift effects than do CHB and JPT^45^, and hence has more alleles per CNV locus (1.66 alleles per CNV locus). A relatively high level of polymorphisms in MEX among non-African populations probably arise from an admixture of individuals with multiple distinct ancestries, which is consistent with previous explanations^37^,^49^.

Because both mutation and migration reduce population genetic divergence^50^, the combined patterns of genetic divergence at CNV and SNP loci provide us with an opportunity to address their relative roles. Previous reports^51^ indicate that the mutation rates are about 1.7 × 10^−6^ to 1.0 × 10^−4^, about 100~10000 times of the point mutation rate at SNP sites (1.8–2.5 × 10^−8^). Fu *et al*.^29^ indicates that the mutation rate for most CNV loci is about order of 10^−5^ per CNV locus per generation. On average, a mutation rate of the order 10^−5^ at the 856 CNV loci could be inferred from the estimate of the population-scaled mutation rate *θ* (=4 *Nμ*) = 0.1415 ± 0.0144^23^, given *N* ~ 3000^45^. Patterns of the *μ*_*c*_/*m*estimates suggest a dominant role that the mutation process plays in shaping population genetic divergence at CNV loci, especially in the non-African populations (Table 5). The low *μ*_*c*_/*m* in African populations could likely arise from their closer genetic relationships where the inter-population gene exchanges are historically more frequent or from natural evolutionary convergence where their genetic compositions become similar since ancestral populations. However, statistical tests indicate that the mutation-drift process can explain the variation at CNV loci in African populations, implying that the latter process could be the main reason for low genetic divergence^23^.

In comparison with the previous results (*G*_*st*_ ~ 0.11) at a few CNV loci^10^ (67 CNV loci and *n* = 270 in total) or at the locus of a specific gene CCL4L^32^ in four HapMap populations (YRI, CEU, and CHB + JPT), our investigation shows much lower population genetic divergence at the 856 CNV loci among these four populations (mean *G*_*st*_ = 0.0345 ± 0.0158; Table 2). This result indicates that the CNV loci shared among 1184 healthy individuals exhibit smaller population genetic divergence. Also, compared with the pairwise *F*_*st*_ across chromosomes at the genome-wide SNP sites (Fig. 2 in Baye^8^), a similarity in pattern at the genome-wide CNV loci exists (Figure S1). The difference is the presence of low population genetic divergence at CNV loci.

A caveat in the above inferences is that it is based on the assumption of equilibrium among the processes of mutation, drift, and migration at CNV and SNP loci in human populations. Like conventional population genetics analyses in different organisms, such an equilibrium might not be attained in reality, and a dynamic model of evolution is more realistic for further investigation. However, concerning the estimates of 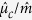, the qualitative conclusion about the major effects of mutation on population genetic divergence cannot be rejected at the genome-wide CNV loci^29^, especially in non-African populations.

Although small LDs are difficult to detect owing to the statistical power, very few CNV loci exhibit significant gametic and zygotic LDs from either the same or different chromosomes. This is different from the patterns at the genome-wide SNP sites (Hu and Hu^37^ for zygotic LDs with the recombination rate <10%, Reich *et al*.^36^ for gametic LDs with the recombination rate <16%, and Koch *et al*.^52^ for gametic LDs with the recombination rate >25%). The CNV loci on the same chromosomes (except a few overlapped loci) are distributed over a wide range of distances, with an average recombination rate of 3.3% (0~35%). The significant correlations among CNV loci do not exist across populations^53^. The generally concordant pattern of no significant gametic and zygotic LDs provides no evidence for the presence of functionally epistatic CNV loci^26^,^27^, different from the results at genome-wide SNP sites^37^.

Patterns of LDs also suggest that the effects of mutation on reducing LDs are stronger than the effects of migration that increases LDs. The gametic LDs at CNV loci gradually decay with time in African populations, and the same is the case for the zygotic LDs at CNV loci^53^, except for the overlapped CNV loci (but not for one pair of CNV loci on Chr 7 with a physical distance of 2658 bp that requires a longer time to decay). The gametic LDs at CNV loci initially formed by the founder effects in non-African populations also decay with time due to the mutation and recombination effects. The same is the case for the zygotic LDs^38^. If recombination is the dominant process in eroding LDs, a certain proportion of CNV loci could maintain significant LD within very short distances except for overlapped loci. Such an expected pattern is not observed (Tables S4 and S5). High mutation rates causing low LDs between CNV and SNP loci are also discussed^54^. Thus, the mutation effects could be greater than the recombination effects in eroding both gametic and zygotic LDs although recombination and mutation effects are both involved in reducing LDs^55^.

Finally, our investigation suggests differential evolutionary processes at CNV and SNP loci along chromosomes. Although mosaic patterns occur in genome architecture in terms of different measures of genetic diversity or from different perspectives^53^, the DNA segments with CNV loci themselves display individual blocks each with a small level of population genetic divergence. These blocks are different from the gametic or zygotic LD blocks at SNP sites since recombination within CNV loci should rarely occur. The LD blocks between CNV loci cannot be maintained due to the effects of the high mutation rates.

## Methods

### Genotype data at CNV loci

Genotype data in 11 HapMap Phase III populations, released by The International HapMap 3 Consortium, was downloaded from ftp://ftp.ncbi.nlm.nih.gov/hapmap/cnv_data/hm3_cnv_submission.txt. The data differs from most accessible data sets in that it provides the discrete copy numbers per CNV locus. The copy numbers at a CNV locus are derived through a two-step process according to Altshuler *et al*.^31^ The first step is to detect copy number variation on each chromosome by analyzing the probe-level intensity data from both the Affymetrix and Illumina arrays. QuantiSNP^56^ and Birdseye^57^ algorithms are used to identify CNV loci separately. Common CNV loci are further identified, and refined to ensure qualified copy number variant calls. The second step is to determine the discrete copy numbers for each CNV locus from the probe-level intensity data. CNVtools^33^ and a two-dimensional model (Gaussian mixture)^31^, are used to infer the copy numbers from the maximum posterior likelihood function. A meta-approach combining the two algorithms and other criteria are used to further refine the discrete copy number classes to ensure reliable copy number estimates per diploid genomes. This second step for estimating the copy number per CNV locus is not conducted in most archived CNV data sets although later techniques for CNV locus detection are now more advanced.

Diploid genotypes were recorded in integers (0, 1, 2, 3, and 4): 0 for the genotype without any allele copy in both gametes, 1 for the genotype with one allele copy in one gamete but without any copy in the other gamete, 2 for the genotype with one allele copy in each gamete, 3 for the genotype with one allele copy in one gamete and two allele copies in the other gamete, and 4 for the genotype with two allele copies in each gamete. From the individual IDs in the HapMap project, eleven populations were extracted from the pooled data (hm3_cnv_submission.txt): ASW (African ancestry in Southwest USA), CEU (Utah residents with Northern and Western European ancestry from the CEPH collection), CHB (Han Chinese in Beijing, China), CHD (Chinese in Metropolitan Denver, Colorado), GIH (Gujarati Indians in Houston, Texas), JPT (Japanese in Tokyo, Japan), LWK (Luhya in Webuye, Kenya), MEX (Mexican ancestry in Los Angeles, California), MKK (Maasai in Kinyawa, Kenya), TSI (Toscans in Italy), and YRI (Yoruba in Ibadan, Nigeria). Sample size for each population is shown in Table 1. The number of CNV loci per Chr ranges from 11 on Chr 22 to 68 on Chr 2, with 856 common CNV loci in total. Mean size of CNV loci per Chr is ~0.02 Mb, ranging from 26 to 456897 bp. The physical distance between adjacent CNV loci per Chr is ~3.3 Mb on average, ranging from 0 (partially overlapped loci) to 34804235 bp. There are 29 CNV loci that are partially overlapped on chromosomes.

### Allele frequency

Because the maximum number of allele copies is four at a CNV locus in the diploid genotype dataset of HapMap Phase III populations, a three-allele system is used to describe the genotype composition. Note that a system of more than three alleles is needed if the number of allele copies is more than 4 in a diploid genotype^23^,^58^. Let *A*_0_, *A*_1_, and *A*_2_ be the alleles with 0-, 1-, and 2-copies at a CNV locus, respectively. Allele *A*_1_ may be the most abundant variant in a population (the segment on the reference genome), while alleles *A*_0_ and *A*_2_ are likely less abundant at a CNV locus. Owing to lack of information needed to separate distinct genotypes with the same copy numbers in diploids, allele frequencies under Hardy-Weinberg equilibrium (HWE) were estimated using the expectation-maximization (EM)^23^,^29^,^59^,^60^. Polymorphism was measured in terms of the number of observed alleles per CNV locus (*N*_*a*_), the percentage of polymorphic loci, *P*(99%), and the genetic diversity in a population (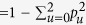 where *p*_*u*_ is the *u*th allele frequency) which is equal to the expected heterozygosity (*H*_e_) under HWE.

### Genetic divergence

Population genetic differentiation was measured by *G*_*st*_^44^: *G*_*st*_ = 1 − *H*_*s*_/*H*_*t*_ where *H*_s_ is the mean of the expected heterozygosity (*H*_*e*_) per locus over all populations and *H*_*t*_ is the expected heterozygosity per locus in the pooled population. The 95% confidence intervals (CIs) for *G*_*st*_ was derived using the bootstrapping approach. To relate the population genetic differentiation to the time since the populations diverge from a single ancestral population, genetic distance was measured^46^. This distance develops under a specific evolutionary processes. Nei’s genetic distance^44^ was used to measure population genetic divergence: *D* = −ln(*I*) where 
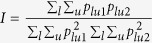
 in which *p*_*lu*1_ and *p*_*lu*2_ are the frequencies of alleles *u*1 and *u*2 at the *l*th locus from populations 1 and 2, respectively. Under the neutral process (mutation and genetic drift), Nei’s genetic distance is linearly related to the time since divergence (*t*), i.e. 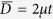
44,46, and its approximate variance *V(t*) = *V(D*)/4*μ*^2^, given a mutation rate *μ*. Standard deviations for *G*_*st*_ and Nei’s genetic distance were calculated using the jackknife method^46^.

### LD tests

To assess the properties of CNV loci relevant for interpreting population genetic divergence, both the gametic and zygotic LDs were tested in each population. Assuming that CNV loci are involved in fitness, a comparison of gametic LD with the maximum zygotic LD in value can be used to determine whether epistasis occurs or not among loci^37^,^38^,^39^. If the maximum zygotic LD (high order LD) is greater than the gametic LD (low order) in value, epistasis exists between loci, which otherwise does not occur (additive or neutral effects). This relationship has been applied to analyzing genome-wide SNP sites^37^, providing the evidence of epistasis among many intron SNP sites in each of the 11 populations. For a pair of CNV loci each with three alleles, there are 9 types of two-non-allele gametes. Let *d*_*ij*_ (*i, j* = 0, 1, 2) be the gametic LD between allele *i* at the first locus and allele *j* at the second locus, and *p*_*ij*_ be the gametic frequency in the population. MLE of the frequency of a genotype pair, 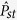
(*s, t* = 0, 1, 2, 3, 4), can be obtained using the direct counting method. An EM method is used to estimate the gametic frequency through an iterative calculation, which is described below:


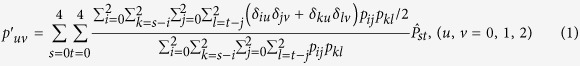


where *δ*_*ij*_, a Kronecker delta variable, is equal to 1 when *i* = *j*, and 0 when *i*≠*j*. Note that the E- and M-steps are combined into one formula in equation (1). Thus, given the initial gametic frequency *p*_*ij*_ (*i, j* = 0, 1, 2), the gametic frequency at the next step *p*′_*uv*_ can be calculated using equation (1). Then, replace *p*_*ij*_ in equation (1) with *p*′_*uv*_ and recalculate *p*′_*uv*_ at the next step. This iterative calculation is repeated until the convergence of gametic frequencies is attained.

The gametic LD, *d*_*ij*_, is then estimated as 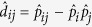 where 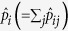 and 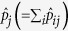 are the MLEs of the frequencies of allele *i* at the first locus and allele *j* at the second locus, respectively. A chi-square statistic with 1 degree of freedom (df) is used to test H_0_: *d*_*ij*_ = 0 46, i.e.


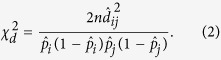


R-square, 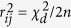, is used to measure gametic LD, which ranges from 0 to 1. Appendix S1 gives the power calculation for the gametic LD test. The power tends to a concave upward curve as the allele frequency increases because the variance 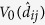 under H_0_ or 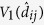 under H_1_ (*d*_*ij*_ ≠ 0) has a maximum value at the intermediate allele frequencies. A large variance increases the uncertainty and hence reduces the power, given a sample size (*n*), a significance level (*α*), and gametic LD. The power also increases as the sample size or the gametic LD increases.

Let *D*_*ij*_ be the zygotic LD between genotypes *i* at the first locus and *j* at the second locus (*i, j* = 0, 1, 2, 3, 4) in the population. The MLE of zygotic LD, 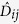, from the sample of size *n* can be obtained by 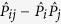
where 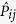 is the MLE of the joint frequency of genotypes *i* at the first locus and *j* at the second locus, and 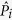 (or 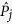) is the frequency of genotype *i* (or *j*). To test H_0_: *D*_*ij*_ = 0, a chi-square statistic with 1 df is set as


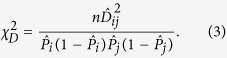


The normalized r-square is set as 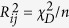, which ranges from 0 to 1^37^,^39^,^61^. Appendix S2 derives the power calculation for the zygotic LD test. Similarly, the power increases as the sample size or the zygotic LD increases. The power may be relatively lower for testing zygotic LD than for testing gametic LD due to the doubling of sample size in gametic LD tests.

The significance tests of gametic and zygotic LDs were conducted at the genome-wide level in each population, and hence a Bonferroni adjusted p-value was set as 0.05/the number of all pairs of CNV loci across 22 chromosomes, ranging from 1.88 × 10^−7^~6.91 × 10^−7^ owing to different numbers of polymorphic loci in the 11 populations. To minimize the impacts of minor allele frequency (MAF) on amplifying gametic LD test or on increasing false-positive errors, those alleles with their frequencies being out of the range [0.05, 0.95] in the samples were excluded in testing gametic LD. For the same reason, those genotypes with genotypic frequencies beyond the range [0.05, 0.95] in the samples were excluded in testing zygotic LD. Sample sizes ranging from 77 to 171 can provide appropriate statistical power for genotypic frequencies within the range [0.05, 0.95] (Appendix S2). Since the constraints 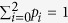 and 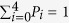 hold, only four gametic LDs and sixteen zygotic LDs were tested for each pair of CNV loci. Note that CNV loci were not filtered out by frequency except in this LD analysis.

### Joint mutation and migration rates

Consider a neutral CNV locus with three alleles. Let *μ*_*c*_ be the mutation rate of one allele to any of the other two alleles at a CNV locus. The probability density distribution (pdf) for the allele frequency under an equilibrium among genetic drift, mutation, and migration effects can be approximated by synthesizing Kimura’s^19^ and Wright’s^50^ work, i.e.


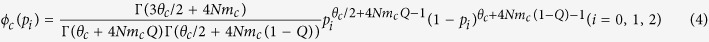


where *N* is the effective population size, *m*_*c*_ is the migration rate per generation for an allele at a CNV locus, *Q* is the migrant allele frequency, and *θ*_c_ (aka “population diversity”) is the population-scaled mutation rate (=4*Nμ*_c_). *F*_*st*_ per locus is derived as


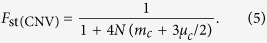


The practical population differentiation with *F*_*st*_^62^ is measured by *G*_*st*_^44^ for a three-allele locus.

Similarly, the pdf of allele frequency at a bi-allelic SNP locus under an equilibrium among genetic drift, mutation, and migration effects can be approximated by synthesizing Kimura’s^19^ and Wright’s^50^ work,


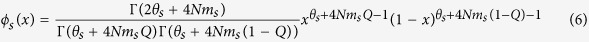


where *m*_*s*_ is the migration rate per generation, *Q* is the migrant allele frequency, and *θ*_s_ is equal to 4*Nμ*_s_ in which *μ*_s_ is the mutation rate at an SNP locus. *F*_*st*_ per locus is derived as


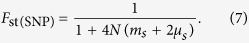


The relative extent of genetic divergence at the genome-wide SNP sites versus at the genome-wide CNV loci is measured by the ratio of *F*_*st(SNP*)_/*G*_*st(CNV*)_, and its standard deviation can be estimated from the variance approximation:


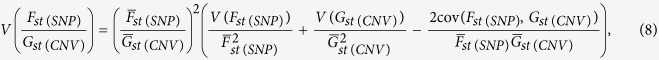


where 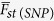 and 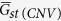 are the means of *F*_*st*(SNP)_ and *G*_*st*(CNV)_, respectively, and *cov(F*_*st*(SNP)_, *G*_*st*(CNV)_) is the covariance between *F*_*st(SNP)*_ and *G*_*st(CNV)*_. The above expression is derived by the delta method^63^. Estimate of the variance of the ratio can be approximated by assuming that the covariance, cov(*F*_*st*(SNP)_, *G*_*st*(CNV)_) is negligible at the genome-wide scale. Correlations between CNV and SNP loci are weak, which could arise from the effects of transposition events, recurrent mutation/reversions, or the preference of CNV loci at the low density of SNP sites on chromosomes^12^,^54^.

From equations (5) and (7), the ratio of the joint migration and nutation rates at CNV loci to those at SNP sites is estimated as


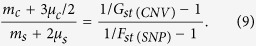


Similarly, the variance of this ratio can be estimated using the delta method^51^. Let *X* = *F*_*st(SNP*)_(1 − *G*_*st(CNV*)_) and *Y* = *G*_*st(CNV*)_(1 − *F*_*st(SNP*)_). Again, assume that cov(*F*_*st(SNP)*_, *G*_*st(CNV)*_) is neglected at the genome-wide scale. The variance *V(X*) is given by


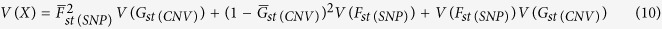


*V(Y*) can be obtained by replacing *F*_*st(SNP)*_ and 1 − *G*_*st(CNV)*_ in equation (10) with *G*_*st(CNV)*_ and 1 − *F*_*st(SNP)*_, respectively. Similarly, *V(XY*) can be obtained by replacing 1 − *G*_*st(CNV)*_ in equation (10) with *G*_*st(CNV*)_. The covariance cov(*X, Y*) is given by


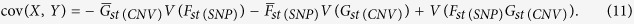


The variance of the ratio *V(X*/*Y*) can be estimated from the following expression,


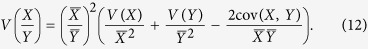


The variance 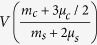 can be appropriately estimated by *V(X*/*Y*) in equation (12), especially when the sample sizes are large.

It is appropriate to assume that the migration rate is the same, on average, at the neutral CNV and SNP loci (*m*_*c*_ = *m*_*s*_ = *m*) although local variation might occur among loci (e.g., due to the genetic hitchhiking effects). Also, compared with the migration rate, the point mutation rate at the SNP sites can be neglected. Thus, the ratio of the mutation rate to the migration rate at CNV loci can be estimated:


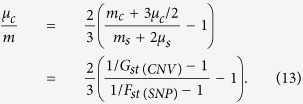


The standard deviation of the *μ*_*c*_/*m* estimate can be obtained according to equation (12), i.e.


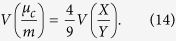


## Additional Information

**How to cite this article**: Hu, X.-S. *et al*. High mutation rates explain low population genetic divergence at copy-number-variable loci in *Homo sapiens. Sci. Rep.* **7**, 43178; doi: 10.1038/srep43178 (2017).

**Publisher's note:** Springer Nature remains neutral with regard to jurisdictional claims in published maps and institutional affiliations.

## Supplementary Material

Supplementary Dataset 1

Supplementary Dataset 2

Supplementary Dataset 3

Supplementary Tables, Appendices and Figure S1

## Figures and Tables

**Table 1:** Sample sizes and polymorphisms at the genome-wide CNV loci in 11 human populations.

Populations	Sample sizes	*P*(99%)*	*N*_a_	Mean *H*_e_ ± *S*_d_
ASW	83	85.16	1.90	0.1290 ± 0.1457
CEU	165	57.71	1.62	0.1123 ± 0.1622
CHB	84	46.14	1.50	0.1072 ± 0.1651
CHD	85	46.61	1.50	0.1047 ± 0.1636
GIH	88	53.85	1.58	0.1121 ± 0.1629
JPT	86	44.51	1.48	0.1079 ± 0.1669
LWK	90	80.49	1.85	0.1268 ± 0.1483
MEX	77	62.38	1.66	0.1130 ± 0.1599
MKK	171	83.53	1.88	0.1248 ± 0.1522
TSI	88	56.78	1.60	0.1123 ± 0.1626
YRI	167	80.14	1.84	0.1322 ± 0.1496

^*^P(99%): the percentage of polymorphic loci where the frequency of the most common allele was ≤0.99.

*N*_a_: the number of observed alleles per CNV locus; *H*_*e*_: the expected heterozygosity under Hardy-Weinberg equilibrium.

**Table 2:** Comparison of the pairwise *G*_*st*(CNV)_ at the genome-wide CNV loci with the pairwise *F*_*st*(SNP)_ at the genome-wide SNP sites^7^.

	ASW	CEU	CHB	CHD	GIH	JPT	LWK	MEX	MKK	TSI	YRI
ASW		0.0275 (0.0001)	0.0357 (0.0001)	0.0353 (0.0001)	0.0267 (0.0001)	0.0336 (0.0001)	0.0071 (0.00001)	0.0258 (0.0001)	0.0087 (0.00001)	0.0255 (0.00005)	0.0061 (0.00001)
CEU	3.7081 (0.0230)		0.0307 (0.0001)	0.0307 (0.0001)	0.0140 (0.00004)	0.0331 (0.0001)	0.0348 (0.0001)	0.0116 (0.00003)	0.0248 (0.00005)	0.0038 (0.00001)	0.0375 (0.0001)
CHB	3.9697 (0.0210)	3.5964 (0.0251)		0.0038 (0.00001)	0.0289 (0.0001)	0.0063 (0.00002)	0.0413 (0.0001)	0.0246 (0.0001)	0.0295 (0.0001)	0.0332 (0.0001)	0.0401 (0.0001)
CHD	4.0432 (0.0213)	3.6545 (0.0253)	0.2649 (0.0265)		0.0280 (0.0001)	0.0073 (0.00003)	0.0415 (0.0001)	0.0243 (0.0001)	0.0295 (0.0001)	0.0326 (0.0001)	0.0403 (0.0001)
GIH	3.5497 (0.0200)	2.4946 (0.0227)	2.6315 (0.0221)	2.7458 (0.0229)		0.0304 (0.0001)	0.0333 (0.0001)	0.0147 (0.00004)	0.0221 (0.00005)	0.0139 (0.00004)	0.0328 (0.0001)
JPT	3.9680 (0.0209)	3.3981 (0.0234)	1.1119 (0.0163)	1.0957 (0.0144)	2.5390 (0.0181)		0.0421 (0.0001)	0.0260 (0.0001)	0.0306 (0.0001)	0.0352 (0.0001)	0.0406 (0.0001)
LWK	1.4017 (0.0142)	4.1900 (0.0242)	4.2389 (0.0187)	4.2400 (0.0210)	3.9617 (0.0195)	4.1856 (0.0207)		0.0331 (0.0001)	0.0082 (0.00002)	0.0332 (0.0001)	0.0059 (0.00001)
MEX	3.6383 (0.0206)	2.6817 (0.0118)	2.0810 (0.0217)	2.9173 (0.0221)	2.3857 (0.0152)	2.6898 (0.0205)	4.0130 (0.0196)		0.0214 (0.0001)	0.0112 (0.00003)	0.0330 (0.0001)
MKK	1.6680 (0.0576)	4.1655 (0.0217)	4.8353 (0.0256)	4.8624 (0.0258)	4.2751 (0.0243)	4.7094 (0.0248)	2.0688 (0.0129)	4.4724 (0.0255)		0.0206 (0.00004)	0.0128 (0.00002)
TSI	3.8807 (0.0210)	1.0415 (0.0262)	3.3390 (0.0231)	3.4390 (0.0236)	2.4423 (0.0159)	3.1983 (0.0219)	4.2654 (0.0225)	2.8533 (0.0195)	4.7502 (0.0307)		0.0334 (0.0001)
YRI	1.5206 (0.0662)	4.1929 (0.0203)	4.6181 (0.0199)	4.6174 (0.0199)	4.3656 (0.0230)	4.5913 (0.0242)	1.3481 (0.0171)	4.3467 (0.0230)	2.1023 (0.0087)	4.5808 (0.0201)	

The above diagonal values are the mean multilocus *G*_*st*(CNV)_ estimates, and the below diagonal values are the ratios of *F*_*st*(SNP)_/*G*_*st*(CNV)_. Standard deviations are shown in parentheses.

**Table 3:** Means and standard deviations of significant gametic LDs (r-squares) in 11 human populations*.

	ASW	CEU	CHB	CHD	GIH	JPT	LWK	MEX	MKK	TSI	YRI
*d*_00_	0.031%	0.053%	0.045%	0.054%	0.034%	0.041%	0.027%	0.044%	0.034%	0.031%	0.045%
	0.84 ± 0.32	0.78 ± 0.32	0.79 ± 0.33	0.72 ± 0.35	0.75 ± 0.34	0.76 ± 0.34	0.80 ± 0.34	0.79 ± 0.33	0.68 ± 0.43	0.86 ± 0.24	0.64 ± 0.44
	21(16:5)	28(17:11)	26(16:10)	30(16:14)	27(16:11)	25(15:10)	21(15:6)	28 (17:11)	27(16:11)	25(17:8)	27(15:12)
	0.21 ± 0.04	0.09 ± 0.02	0.18 ± 0.03	0.17 ± 0.01	0.19 ± 0.02	0.18 ± 0.02	0.18 ± 0.04	0.21 ± 0.04	0.10 ± 0.02	0.18 ± 0.03	0.10 ± 0.02
	59	37	9	13	9	5	42	34	60	11	77
*d*_01_	0.035%	0.058%	0.050%	0.062%	0.037%	0.047%	0.032%	0.048%	0.038%	0.033%	0.050%
	0.79 ± 0.32	0.78 ± 0.31	0.80 ± 0.32	0.74 ± 0.34	0.80 ± 0.30	0.75 ± 0.33	0.80 ± 0.33	0.80 ± 0.39	0.65 ± 0.42	0.85 ± 0.23	0.63 ± 0.43
	23(18:5)	30(19:11)	28(18:10)	32(18:14)	27(18:9)	28(17:11)	23(17:6)	29(19:10)	28(16:12)	26(18:8)	29(17:12)
	0.21 ± 0.04	0.10 ± 0.01	0.20 ± 0.03	0.17 ± 0.01	0.19 ± 0.03	0.18 ± 0.02	0.19 ± 0.04	0.21 ± 0.04	0.10 ± 0.02	0.17 ± 0.03	0.10 ± 0.02
	68	41	11	17	12	6	52	40	69	13	89
*d*_10_	0.034%	0.064%	0.057%	0.062%	0.044%	0.051%	0.029%	0.048%	0.040%	0.041%	0.051%
	0.82 ± 0.32	0.75 ± 0.34	0.75 ± 0.34	0.71 ± 0.35	0.76 ± 0.33	0.74 ± 0.34	0.77 ± 0.36	0.78 ± 0.32	0.68 ± 0.42	0.83 ± 0.26	0.64 ± 0.44
	23(17:6)	31(18:13)	27(16:11)	31(16:15)	28(17:11)	27(16:11)	22(15:7)	29(18:11)	27(16:11)	27(18:9)	27(15:12)
	0.22 ± 0.09	0.13 ± 0.17	0.27 ± 0.23	0.21 ± 0.16	0.23 ± 0.15	0.26 ± 0.26	0.18 ± 0.04	0.23 ± 0.11	0.10 ± 0.02	0.21 ± 0.14	0.11 ± 0.07
	68	47	17	18	19	10	47	40	76	21	92
*d*_11_	0.041%	0.071%	0.064%	0.073%	0.048%	0.059%	0.034%	0.056%	0.045%	0.047%	0.058%
	0.79 ± 0.32	0.74 ± 0.34	0.74 ± 0.35	0.72 ± 0.34	0.80 ± 0.30	0.74 ± 0.33	0.78 ± 0.34	0.82 ± 0.30	0.64 ± 0.44	0.82 ± 0.27	0.65 ± 0.43
	28(19:9)	34(20:14)	31(19:13)	35(19:16)	29(19:10)	31(18:13)	25(17:8)	30(20:10)	29(16:13)	30(19:11)	33(18:15)
	0.22 ± 0.08	0.13 ± 0.34	0.26 ± 0.21	0.20 ± 0.12	0.22 ± 0.30	0.24 ± 0.24	0.19 ± 0.04	0.22 ± 0.09	0.10 ± 0.01	0.20 ± 0.13	0.11 ± 0.07
	80	53	19	23	22	12	55	49	85	25	104

^*^The percentages in the same row as *d*_*ij*_ (*i, j = 0, 1*) in the table are the proportions of significant gametic LDs among all pairs of LD tests. The data in the second row under each *d*_*ij*_ is the gametic LD among CNV loci from the same chromosomes. The data in the third row under each *d*_*ij*_ is the observed numbers of pairs with significant LDs from the same chromosomes (non-overlapped locus pairs: overlapped locus pairs). The data in the fourth row under each *d*_*ij*_ is the significant gametic LDs among CNV loci from different chromosomes. The data in the fifth row under each *d*_*ij*_ is the observed numbers of pairs with significant LDs among CNV loci from different chromosomes.

**Table 4:** Percentages of the pairs of CNV loci with significant zygotic LDs in 11 human populations*.

LD	ASW	CEU	CHB	CHD	GIH	JPT	LWK	MEX	MKK	TSI	YRI
*D*_00_	0.0045	0.0082	0.0141	0.0189	0.0123	0.0193	0.0046	0.0063	0.0039	0.0093	0.0067
*D*_01_	0.0041	0.0066	0.0077	0.0101	0.0047	0.0083	0.0025	0.0042	0.0031	0.0059	0.0034
*D*_02_	0.0019	0.0033	0.0026	0.0025	0.0019	0.0028	0.0013	0.0021	0.0008	0.0025	0.0009
*D*_03_	0.0008	0.0016	0.0026	0.0025	0.0019	0.0028	0.0008	0.0014	0.0008	0.0017	0.0009
*D*_10_	0.0041	0.0115	0.0116	0.0126	0.0075	0.0124	0.0038	0.0063	0.0043	0.0085	0.0051
*D*_11_	0.0143	0.0271	0.0308	0.0327	0.0226	0.0332	0.0143	0.0253	0.0129	0.0221	0.0111
*D*_12_	0.0113	0.0222	0.0218	0.0214	0.0151	0.0276	0.0114	0.0211	0.0125	0.0161	0.0098
*D*_13_	0.0023	0.0025	0.0026	0.0025	0.0019	0.0041	0.0008	0.0014	0.0027	0.0017	0.0013
*D*_20_	0.0034	0.0057	0.0051	0.0050	0.0029	0.0069	0.0008	0.0028	0.0008	0.0042	0.0013
*D*_21_	0.0143	0.0246	0.0244	0.0264	0.0160	0.0304	0.0105	0.0232	0.0129	0.0178	0.0111
*D*_22_	0.0139	0.0279	0.0283	0.0252	0.0160	0.0359	0.0127	0.0246	0.0145	0.0187	0.0128
*D*_23_	0.0023	0.0016	0	0	0	0.0014	0.0004	0	0.0020	0	0.0013
*D*_30_	0.0004	0.0025	0	0.0013	0	0	0	0.0007	0	0	0
*D*_31_	0.0026	0.0049	0	0.0038	0.0019	0.0069	0.0017	0.0035	0.0024	0.0025	0.0026
*D*_32_	0.0041	0.0049	0.0051	0.0038	0.0047	0.0069	0.0021	0.0049	0.0016	0.0034	0.0034
*D*_33_	0.0015	0	0	0	0	0.0014	0.0008	0.0007	0.0004	0.0008	0.0013

^*^D_*ij*_ is the zygotic LD between genotype *i* at the first locus and *j* at the second locus (*i, j* = 0, 1, 2, 3).

**Table 5:** Ratios of the joint mutation and migration rates at CNV loci to those at SNP sites (above diagonal), and the ratios of the mutation rate to the migration rate at CNV loci (below diagonal).

	ASW	CEU	CHB	CHD	GIH	JPT	LWK	MEX	MKK	TSI	YRI
ASW		4.008 (0.0303)	4.4667 (0.0288)	4.5564 (0.0293)	3.8132 (0.0266)	4.8110 (0.0312)	1.4126 (0.0144)	3.9084 (0.0278)	1.6765 (0.0587)	4.1896 (0.0250)	1.5129 (0.0664)
CEU	2.0054 (0.0202)		3.9223 (0.0309)	3.9942 (0.0311)	2.5468 (0.0239)	3.6992 (0.0284)	4.7303 (0.0335)	2.7259 (0.0115)	4.5349 (0.0262)	1.0528 (0.0266)	4.7910 (0.0286)
CHB	2.3111 (0.0192)	1.9482 (0.0206)		0.2624 (0.0263)	2.7677 (0.0256)	1.1119 (0.0164)	4.9274 (0.0269)	2.9478 (0.0260)	5.7238 (0.0375)	3.6286 (0.0282)	5.4445 (0.0289)
CHD	2.3709 (0.0195)	1.9962 (0.0207)	—*		2.8878 (0.0266)	1.0967 (0.0145)	4.9298 (0.0299)	3.0640 (0.0266)	5.5163 (0.0368)	3.7503 (0.0289)	5.4487 (0.0288)
GIH	1.8755 (0.0177)	1.0312 (0.0159)	1.1785 (0.0171)	1.2586 (0.0178)		2.6720 (0.0208)	4.4186 (0.0269)	2.4310 (0.0159)	4.6233 (0.0290)	2.4969 (0.0168)	4.9364 (0.0321)
JPT	2.5407 (0.0208)	1.7994 (0.0189)	0.0746 (0.0109)	0.0644 (0.0097)	1.1147 (0.0139)		4.8732 (0.0294)	2.8197 (0.0244)	5.3380 (0.0352)	3.4744 (0.0264)	5.4210 (0.0350)
LWK	0.2751 (0.0096)	2.4869 (0.0223)	2.6183 (0.0180)	2.6199 (0.0199)	2.2790 (0.0179)	2.5822 (0.0196)		4.4772 (0.0272)	2.0917 (0.0135)	4.7997 (0.0314)	1.3588 (0.0173)
MEX	1.9390 (0.0185)	1.1506 (0.0077)	1.2985 (0.0173)	1.3760 (0.0177)	0.9540 (0.0106)	1.2131 (0.0162)	2.3181 (0.0181)		4.8450 (0.0363)	2.9185 (0.0204)	4.9055 (0.0319)
MKK	0.4510 (0.0391)	2.3566 (0.0175)	3.1492 (0.0250)	3.0109 (0.0246)	2.4155 (0.0194)	2.8920 (0.0235)	0.7278 (0.0090)	2.5633 (0.0242)		5.1655 (0.0365)	2.1402 (0.0088)
TSI	2.1264 (0.0167)	0.0352 (0.0177)	1.7524 (0.0188)	1.8335 (0.0193)	0.9980 (0.0112)	1.6496 (0.0176)	2.5331 (0.0210)	1.2790 (0.0136)	2.7770 (0.0244)		5.2357 (0.0291)
YRI	0.3419 (0.0443)	2.5273 (0.0190)	2.9630 (0.0193)	2.9658 (0.0192)	2.6243 (0.0214)	2.9473 (0.0234)	0.2392 (0.0115)	2.6037 (0.0213)	0.7601 (0.0059)	2.8238 (0.0194)	

^*^negative value.

Standard deviations are shown in parentheses.
