# Supplementary material for: High mutation rates explain low population genetic divergence at copy-number-variable loci in Homo sapiens
Source: Sci Rep. 2017 Feb 22;7:43178. doi: 10.1038/srep43178 (PMC5320550; doi:10.1038/srep43178)

High mutation rates explain low population genetic divergence at copy-number-variable loci in *Homo sapiens*

Xin-Sheng Hu [1, 2*], Francis C Yeh[3], Yang Hu[4], Li-Ting Deng[1,2], Richard A Ennos[5], Xiaoyang Chen[1, 2*]

1. Guangdong Key Laboratory for Innovative Development and Utilization of Forest Plant Germplasm, South China Agricultural University, Guangdong 510642, China

2. College of Forestry and Landscape Architecture, South China Agricultural University, Guangdong 510642, China

3. Department of Renewable Resources, 751 General Service Building, University of Alberta, Edmonton, AB T6G 2H1, Canada

4. Department of Computing Science, University of Alberta, Edmonton, AB T6G 2S4, Canada

5. Institute of Evolutionary Biology, Ashworth Laboratories, School of Biological Sciences, University of Edinburgh, Edinburgh EH 9 3JT, United Kingdom.

* Correspondence: xinsheng@scau.edu.cn; xychen@scau.edu.cn

Running title: Genetic divergence at CNV loci in *Homo sapiens*

Appendix S1. Statistical power for testing gametic linkage disequilibrium (LD)

The relationship between the power 1-β of testing gametic LD $H_0(d_{ij}=0)$ and the allele frequencies can be analytically approximated in terms of the z-score statistic, which is equivalent to the numerical examination in terms of chi-square statistic (Hu and Yeh 2014), i.e.

$$n=\frac{p_i(1-p_i)p_j(1-p_j)+(1-2p_i)(1-2p_j)d_{ij}-d_{ij}^2}{2d_{ij}^2}\left(z_{(1-\alpha/2)}\sqrt{\frac{V_0(\hat{d}_{ij})}{V_1(\hat{d}_{ij})}}+z_{(1-\beta)}\right)^2 \qquad \text{(A1)}$$

where $\Pr\left(z_{\alpha/2}\le(\hat{d}_{ij}-0)/\sqrt{V_0(\hat{d}_{ij})}\le z_{(1-\alpha/2)}\right)=1-\alpha$,

$V_1(\hat{d}_{ij})=\left(p_i(1-p_i)p_j(1-p_j)+(1-2p_i)(1-2p_j)d_{ij}-d_{ij}^2\right)/2n$ and $V_0(\hat{d}_{ij})=p_i(1-p_i)p_j(1-p_j)/2n$. Under H_0, $\hat{d}_{ij}$ follows $N(0,V_0(\hat{d}_{ij}))$; and under H_1: $D_{ij}\neq 0$, $\hat{d}_{ij}$ follows $N(E(\hat{d}_{ij}),V_1(\hat{d}_{ij}))$ where $E(\hat{d}_{ij})=(1-1/2n)d_{ij}$. The power trends to a concave upward curve as the allele frequency changes from 0 to 1 because both $V_0(\hat{d}_{ij})$ and $V_1(\hat{d}_{ij})$ have a maximum value at the intermediate allele frequencies.

Numerical examples show that a large variance increases the uncertainty and hence reduces the testing power, given the sample size (n), a significant level (α), and the gametic LD. The power increases as the sample size increases or as the gametic LD increases.

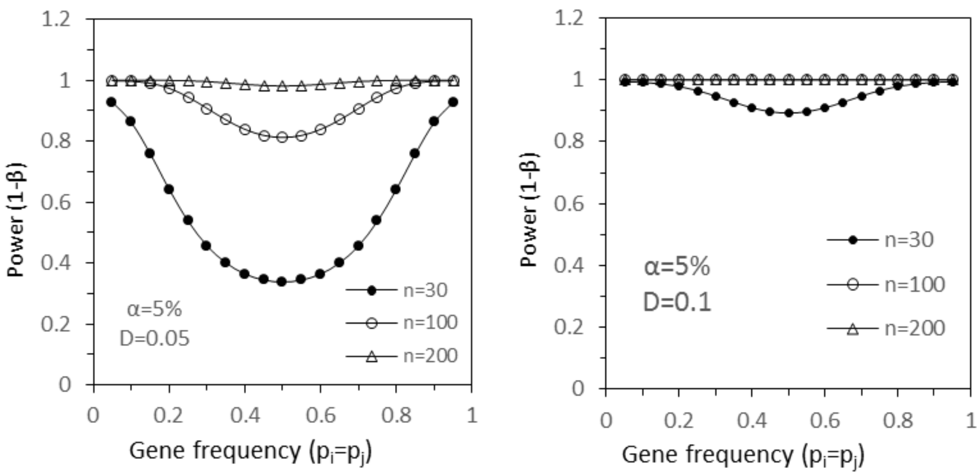

Figure 1. Pattern for the change of the statistical power with the allele frequency. The plot is derived according to equation (A1) by letting $p_i=p_j$ under the case of α=5% and different gametic LDs (D=0.05 and 0.1).

Reference

Hu, X.S., Yeh, F.C. Assessing postzygotic isolation using zygotic disequilibrium in natural hybrid zones. *PloS One* **9**, e100568 (2014).

Appendix S2. Statistical power for testing zygotic linkage disequilibrium (LD)

The sampling variance of zygotic LD, $\hat{D}_{ij}$, is given by

$V(\hat{D}_{ij}) = \left(P_i(1-P_i)P_j(1-P_j) + (1-2P_i)(1-2P_j)D_{ij} - D_{ij}^2\right)/n$ and the expectation is $E(\hat{D}_{ij}) = (1-1/n)D_{ij}$ (Hu and Yeh 2014). Its distribution is $\hat{D}_{ij}$ ~*N*(0, $V_0(\hat{D}_{ij})$) where $V_0(\hat{D}_{ij})$ is $P_i(1-P_i)P_j(1-P_j)/n$ under H_0, or $\hat{D}_{ij}$ ~*N*($E(\hat{D}_{ij})$, $V_1(\hat{D}_{ij})$) where $V_1(\hat{D}_{ij})$ is $V(\hat{D}_{ij})$ under H_1: $D_{ij} \neq 0$. Similarly, the relationship between the testing power 1-*β* and the sample size *n* (or the genotypic frequency) can be numerically examined by the chi-square statistic or analytically approximated by the z-score (Hu and Yeh 2014), i.e.

$$n = \frac{P_i(1-P_i)P_j(1-P_j) + (1-2P_i)(1-2P_j)D_{ij} - D_{ij}^2}{D_{ij}^2}\left(z_{(1-\alpha/2)}\sqrt{\frac{V_0(\hat{D}_{ij})}{V_1(\hat{D}_{ij})}} + z_{(1-\beta)}\right)^2 \qquad \text{(B1)}$$

where $\Pr\left(z_{\alpha/2} \le (\hat{D}_{ij} - 0)/\sqrt{V_0(\hat{D}_{ij})} \le z_{(1-\alpha/2)}\right) = 1-\alpha$. Again, the statistical power trends to a concave upward curve as the genotypic frequency changes from 0 to 1 because both $V_0(\hat{D}_{ij})$ and $V_1(\hat{D}_{ij})$ have a maximum value at the intermediate genotypic frequency. The power also increases as the sample size increases or as the zygotic LD increases. Note that, under the same numerical settings in value between the allele and genotype frequencies, and between gametic and zygotic LDs, the power for testing zygotic LD is relatively lower than that for testing gametic LD due to the doubling of the sample size in the gametic LD analysis.

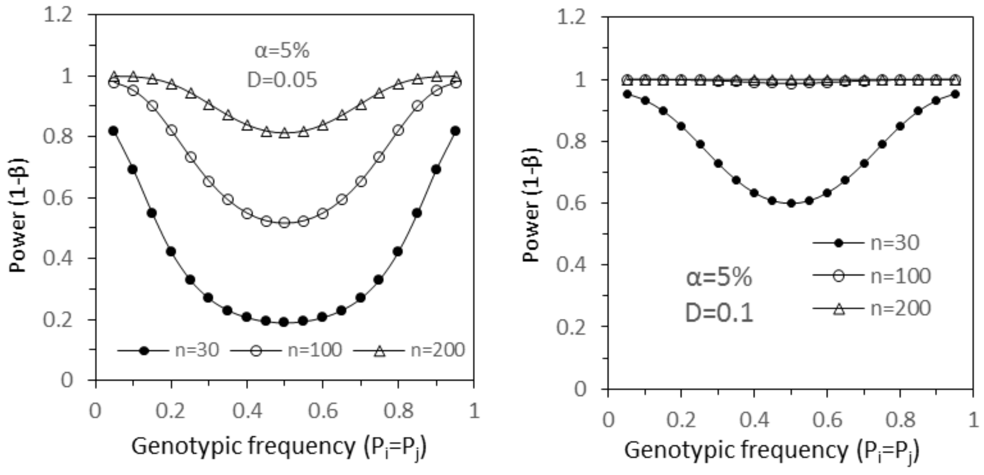

Figure 1. Pattern for the change of the statistical power with the genotypic frequency. The plot is derived according to equation (B1) by letting P_i=P_j under the case of α=5% and different zygotic LDs (D=0.05 and 0.1).

Reference

Hu, X.S., Yeh, F.C. Assessing postzygotic isolation using zygotic disequilibrium in natural hybrid zones. *PloS One* **9**, e100568 (2014).

Table S2. Nei's genetic distances between populations at CNV loci, with their standard deviations in parentheses.

	ASW	CEU	CHB	CHD	GIH	JPT	LWK	MEX	MKK	TSI	YRI
ASW		0.0128 (0.00004)	0.0190 (0.0001)	0.0188 (0.0001)	0.0130 (0.00004)	0.0188 (0.0001)	0.0024 (0.00001)	0.0131 (0.00004)	0.0031 (0.00001)	0.0120 (0.00004)	0.0022 (0.00001)
CEU	0.0128 (0.00004)		0.0139 (0.0001)	0.0141 (0.0001)	0.0056 (0.00002)	0.0150 (0.0001)	0.0174 (0.0001)	0.0047 (0.00002)	0.0119 (0.00004)	0.0012 (0.000004)	0.0198 (0.0001)
CHB	0.0190 (0.0001)	0.0139 (0.0001)		0.0010 (0.000004)	0.0107 (0.00004)	0.0016 (0.00001)	0.0218 (0.0001)	0.0100 (0.00004)	0.0170 (0.0001)	0.0136 (0.00004)	0.0240 (0.0001)
CHD	0.0188 (0.0001)	0.0141 (0.0001)	0.0010 (0.000004)		0.0106 (0.00004)	0.0019 (0.00001)	0.0219 (0.0001)	0.0098 (0.00004)	0.0170 (0.0001)	0.0135 (0.00005)	0.0241 (0.0001)
GIH	0.0130 (0.00004)	0.0056 (0.00002)	0.0107 (0.00004)	0.0106 (0.00004)		0.0114 (0.00004)	0.0166 (0.0001)	0.0058 (0.00002)	0.0119 (0.00004)	0.0051 (0.00002)	0.0186 (0.0001)
JPT	0.0188 (0.0001)	0.0150 (0.0001)	0.0016 (0.00001)	0.0019 (0.00001)	0.0114 (0.00004)		0.0217 (0.0001)	0.0106 (0.00004)	0.0173 (0.0001)	0.0144 (0.0001)	0.0237 (0.0001)
LWK	0.0024 (0.00001)	0.0174 (0.0001)	0.0218 (0.0001)	0.0219 (0.0001)	0.0166 (0.0001)	0.0217 (0.0001)		0.0173 (0.0001)	0.0030 (0.00001)	0.0163 (0.0001)	0.0021 (0.00001)
MEX	0.0131 (0.00004)	0.0047 (0.00002)	0.0100 (0.00004)	0.0098 (0.00004)	0.0058 (0.00002)	0.0106 (0.00004)	0.0173 (0.0001)		0.0122 (0.00004)	0.0040 (0.00001)	0.0196 (0.0001)
MKK	0.0031 (0.00001)	0.0119 (0.00004)	0.0170 (0.0001)	0.0170 (0.0001)	0.0119 (0.00004)	0.0173 (0.0001)	0.0030 (0.00001)	0.0122 (0.00004)		0.0108 (0.00004)	0.0047 (0.00002)
TSI	0.0120 (0.00004)	0.0012 (0.000004)	0.0136 (0.00004)	0.0135 (0.00005)	0.0051 (0.00002)	0.0144 (0.0001)	0.0163 (0.0001)	0.0040 (0.00001)	0.0108 (0.00004)		0.0191 (0.0001)
YRI	0.0022 (0.00001)	0.0198 (0.0001)	0.0240 (0.0001)	0.0241 (0.0001)	0.0186 (0.0001)	0.0237 (0.0001)	0.0021 (0.00001)	0.0196 (0.0001)	0.0047 (0.00002)	0.0191 (0.0001)	

Table S4. The common pairs of CNV loci with significant gametic and zygotic LDs in 11 HapMap populations

Chr	Start	End	Chr	Start	End	Distance(bp)*	Significant LD
Gametic LD							
1	238460638	238461003	1	238460638	238461502	-365	d_{00}, d_{01}, d_{10}, d_{11}
5	103882062	103888503	5	103888272	103888503	-231	d_{00}, d_{01}, d_{10}, d_{11}
5	103882062	103888503	5	103882062	103888948	-6441	d_{00}, d_{01}, d_{10}, d_{11}
5	103882062	103888948	5	103888272	103888503	-676	d_{00}, d_{01}, d_{10}, d_{11}
6	29959422	29973697	6	29959422	30007126	-14275	d_{00}, d_{01}, d_{10}, d_{11}
6	29959422	29973697	6	29959422	30007472	-14275	d_{00}, d_{01}, d_{10}, d_{11}
6	29959422	30007126	6	29959422	30007472	-47704	d_{00}, d_{01}, d_{10}, d_{11}
6	32562253	32679939	6	32605094	32679939	-74845	d_{00}, d_{01}
7	125833260	125833851	7	125836509	125836837	2658	d_{00}, d_{01}, d_{10}, d_{11}
9	29084262	29086271	9	29084549	29087680	-1722	d_{00}, d_{01}, d_{10}, d_{11}
11	42769727	42774615	11	42771111	42774681	-3504	d_{00}, d_{01}, d_{10}, d_{11}
12	11110698	11142827	12	11122722	11142827	-20105	d_{00}, d_{01}, d_{10}, d_{11}
Zygotic LD							
1	238460638	238461003	1	238460638	238461502	-365	D_{00}, D_{11}, D_{22}
5	103882062	103888503	5	103888272	103888503	-231	D_{00}, D_{01}, D_{10}, D_{11}
5	103882062	103888948	5	103888272	103888503	-676	D_{00}, D_{01}, D_{10}, D_{11}
5	103882062	103888503	5	103882062	103888948	-6441	D_{00}, D_{01}, D_{11}
6	32562253	32679939	6	32605094	32679939	-74845	D_{00}, D_{01}
6	32562253	32679939	6	32669797	32705194	-10142	D_{02}, D_{12}
6	32605094	32679939	6	32669797	32705194	-10142	D_{02}, D_{03}, D_{12}
6	29959422	29973697	6	29959422	30007126	-14275	D_{11}, D_{12}, D_{21}, D_{22}
6	29959422	29973697	6	29959422	30007472	-14275	D_{11}, D_{12}, D_{21}, D_{22}
6	29959422	30007126	6	29959422	30007472	-47704	D_{11}, D_{12}, D_{21}, D_{22}
6	32562253	32679939	6	32605094	32679939	-74845	D_{11}
7	125833260	125833851	7	125836509	125836837	2658	D_{00}, D_{01}, D_{22}
7	125832718	125833851	7	125836509	125836837	2658	D_{10}, D_{11}, D_{21}, D_{32}
7	125832718	125833851	7	125833260	125833851	-591	D_{10}, D_{11}, D_{20}, D_{21}, D_{32}
9	29084262	29086271	9	29084549	29087680	-1722	D_{11}
10	70952478	70960784	10	70952492	70960784	-8292	D_{11}, D_{12}, D_{21}
10	81425093	81489917	10	81475459	81489917	-14458	D_{21}
11	42769727	42774615	11	42771111	42774681	-3504	D_{11}, D_{12}, D_{21}
11	54725242	54793048	11	54726437	54738787	-66611	D_{11}, D_{12}, D_{21}, D_{22}
12	11110698	11142827	12	11122722	11142827	-20105	D_{11}, D_{22}
13	71375556	71378557	13	71375556	71387378	-3001	D_{11}, D_{22}
20	62419232	62426597	20	62425139	62426597	-1458	D_{22}

*: The negative values mean that CNV loci are partially overlapped on chromosomes.

Figure S1. Inter-chromosomal variation of pairwise G_{st}s: (a) among all 11 populations where 55 population pairs are plotted in different colors; (b) among four populations (CEU, YRI, CHB, and JPT). The G_{st} shown on each chromosome in (a) and (b) is the mean of the G_{st} values over all CNV loci on that chromosome.

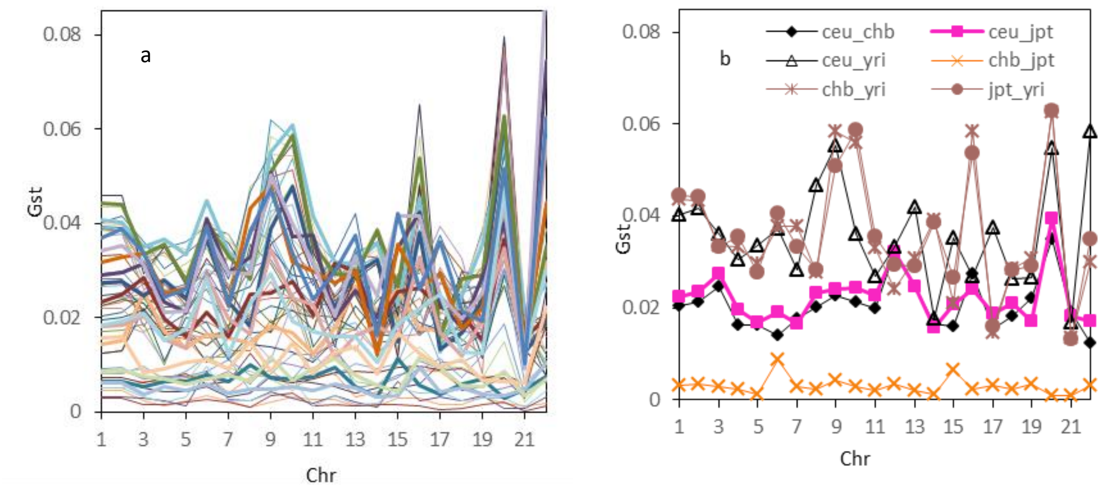

Supplement: Supplementary Tables, Appendices and Figure S1 [file srep43178-s4.pdf]
